# Metal Ions in Neuroscience

**DOI:** 10.1155/MBD.1997.125

**Published:** 1997

**Authors:** C. Ian Ragan

**Affiliations:** Department of Biochemistry and Molecular Biology Neuroscience Research Centre Merck Sharp and Dohme Research Laboratories Terlings Park Harlow CM20 2QR UK

## Abstract

Metal ions are believed to participate in many neurodegenerative conditions. In excitotoxic cell death there is convincing evidence for the participation of Ca^2+^ and Zn^2+^ ions although the exact molecular mechanisms by which these metals exert their effects are unclear. Only in one instance has the metal binding site of metalloenzymes been exploited for therapeutic purposes and this is the use of Li^+^ in the treatment of bipolar affective disorder. Again the exact molecular target is not clear but is likely to involve a Mg^2+^-dependent enzyme of an intracellular signalling pathway. In Parkinson's disease, the selective loss of dopaminergic neurones in the substantia nigra may be caused by radical-mediated damage and there is good evidence to suggest that Fe^2+^ or ^3+^ is important in promoting formation of radical species. The evidence that free radicals are important in mediating other neurodegenerative conditions is less strong but still substantial enough to suggest that removal of reactive oxygen species or preventing their formation may be a valid approach to therapy.


### METAL IONS IN NEUROSCIENCE

C. Ian Ragan

Department of Biochemistry and Molecular Biology, Neuroscience Research Centre,
Merck Sharp and Dohme Research Laboratories, Terlings Park, Harlow CM20 2QR, U.K.

**Abstract**

Metal ions are believed to participate in many neurodegenerative conditions. In excitotoxic cell death there is convincing evidence for the participation of $Ca^{2+}$ and $Zn^{2+}$ ions although the exact molecular mechanisms by which these metals exert their effects are unclear. Only in one instance has the metal binding site of metalloenzymes been exploited for therapeutic purposes and this is the use of $Li^+$ in the treatment of bipolar affective disorder. Again the exact molecular target is not clear but is likely to involve a $Mg^{2+}$-dependent enzyme of an intracellular signalling pathway. In Parkinson's disease, the selective loss of dopaminergic neurones in the substantia nigra may be caused by radical-mediated damage and there is good evidence to suggest that $Fe^{2+}$ or $^{3+}$ is important in promoting formation of radical species. The evidence that free radicals are important in mediating other neurodegenerative conditions is less strong but still substantial enough to suggest that removal of reactive oxygen species or preventing their formation may be a valid approach to therapy.

**Introduction**

In this article, rather than describe the role of metal ions in normal neuronal function, I have focused on abnormal neuronal conditions and the role of metal ions in causing or in preventing them i.e neurotoxic metals and metal-based therapeutics. My reason was principally to reduce the scope of the topic to manageable proportions, but metal ions seem to play an important part in neuronal cell death and a better understanding of their properties could lead to improved therapeutics. Since so many different metals ions are toxic I have also chosen to concentrate on those which have a normal physiological function and which become neurotoxic when this is disturbed.

Many divalent and trivalent metal ions are neurotoxic and there are a variety of mechanisms which contribute or could contribute to cell damage. Probably of greatest importance are effects involving ion channels. Calcium channels are prime targets for blockade by divalent cations[1,2], but the same channels may also transport toxic metals into the cell interior to act on intracellular targets[3]. Ligand-gated ion channels, such as the NMDA receptor, can also act both as targets for metal ions and as transporters[4,5]. There is good evidence that the toxicity of certain metals such as $Zn^{2+}$ for example, is absolutely dependent on transport[6]. The true targets therefore for these (and probably other metals) are intracellular and could in principle involve enzyme inhibition or activation, promotion of free radical formation leading to protein and membrane damage, protein modification and cross-linking etc.

Knowledge of the mechanisms of metal-based toxicity should provide ideas about metal-based therapeutics for CNS disorders. The use of lithium salts in the treatment and prophylaxis of bipolar affective disorder (manic depression) remains, however, the sole example. While the detailed mechanism is not clear at this stage, the idea that lithium ions interfere with intracellular signal transduction pathways seems to have taken root[7]. In the same way that $Zn^{2+}$ toxicity, for example, could result from inhibition of divalent metal- dependent processes in the cell, it seems probable that the therapeutic benefit of $Li^+$ could also derive from an effect on a $Mg^{2+}$-dependent enzyme[7]. The lack of marked selectivity of enzymes for metal ions could explain the toxicity of $Li^+$ at higher concentrations.

An entirely different approach to metal-based drugs also emerges from an understanding of metal-based toxicity. The growing interest in free radical-mediated cell death, in which metal-catalyzed reactions play an important role, has spawned a number of approaches to neuroprotection in response to acute injury or chronic disease. One such is the use of organomanganese complexes which catalyze the dismutation of reactive oxygen species ($O_2^{\cdot-}$, $H_2O_2$)[8]. These compounds may turn out to have utility not only in neurological disorders such as stroke, but also in protecting other tissues subject to ischaemia/reperfusion injury such as the heart.

**Exicitotoxicity, $Ca^{2+}$ and $Zn^{2+}$**

The main excitatory neurotransmitter in the brain is glutamate. The target receptors for glutamate released at glutamatergic synapses are a group of ligand-gated ion channels which permit entry of $Na^{+}$ ions to produce a depolarization of the cell[9]. The family of receptors most involved in normal synaptic transmission are the AMPA/KA group, named after selective agonists for these classes. The NMDA class of glutamate-gated ion channels is blocked by $Mg^{2+}$ in a voltage-dependent manner, i.e. the channels do not function when the cell is polarized[10]. Following interruption to the blood supply to the brain, as in stroke, normal mechanisms regulating the synaptic concentration of glutamate fail[11] (Fig.1).

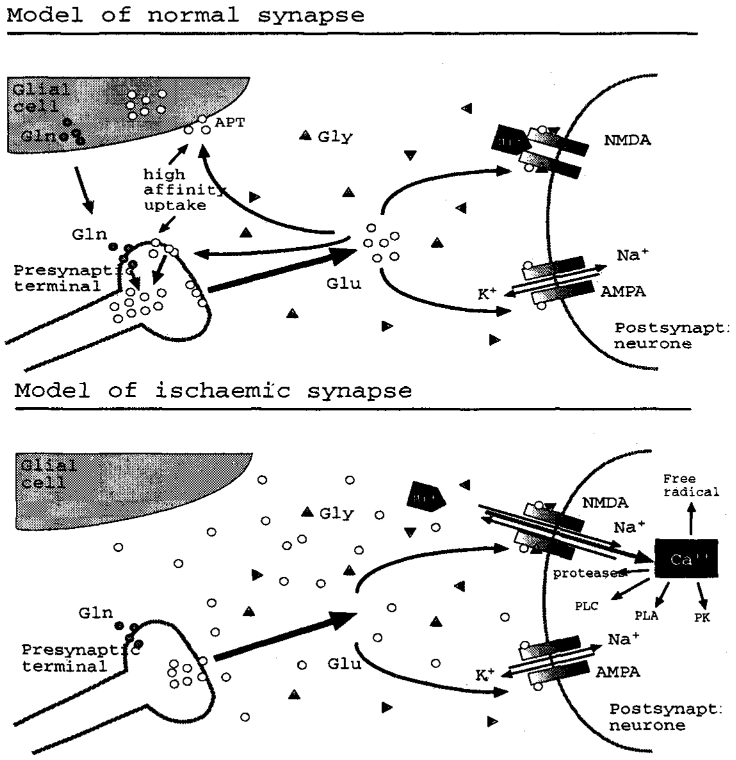

Fig.1. Normal and ischaemic glutamatergic synapses

Energy-dependent processes such as the re-uptake of glutamate into pre-synaptic terminals or neighbouring glial cells cease and the terminal transporter reverses leading to a huge increase in glutamate release. Excessive stimulation of post-synaptic glutamate receptors leads to marked depolarization, and relief of the voltage-dependent block of NMDA receptors. This class, unlike the majority of AMPA/KA receptors, also allows $Ca^{2+}$ entry[12], and under these conditions the excess of intracellular $Ca^{2+}$ overwhelms the buffering capacity of mitochondrial storage and sets in motion a train of events which leads, eventually, to cell death. While $Ca^{2+}$ has been identified as one of the villains for some time, the exact sequence of events is still undefined in detail. $Ca^{2+}$ overload can lead to mitochondrial damage[13] and metabolic failure of the cell even if blood supply is restored. $Ca^{2+}$ also activates proteases such as calpain which may lead to conversion of xanthine dehydrogenase to xanthine oxidase producing potentially toxic superoxide[14]. Cellular swelling occurs as a result of the ion influx. What is clear is that the affected area can be protected, at least in animal models, by blockade of the NMDA receptor and recent clinical data suggest that such a strategy may be effective in man[11].

Recently, another metal ion has been implicated in neuronal damage after transient forebrain ischaemia. It has been known for some time that $Zn^{2+}$ is present in the terminals of central excitatory neurones and is released along with glutamate during normal synaptic transmission. The role of $Zn^{2+}$ is uncertain but it is known that it can act as an inhibitor of post-synaptic NMDA and GABA-A

receptors[4] and voltage-dependent $Ca^{2+}$ channels[1] and therefore its release may be important in limiting cell excitability. $Zn^{2+}$ is also known to be toxic to neurones in culture, and this is mediated by transport into the cell through NMDA receptors or $Ca^{2+}$ channels[3,5]. $Zn^{2+}$ is therefore both an inhibitor of these channels and a substrate for them. *In vivo*, transient forebrain ischaemia in rat leads to translocation of $Zn^{2+}$ into those hippocampal neurones which are most vulnerable, before degeneration sets in, suggesting that this is cause rather than effect[15]. Cell death can be prevented by chelating extracellular $Zn^{2+}$ with CaEDTA showing that intracellular $Zn^{2+}$ is the culprit, as suggested by the *in vitro* experiments[6]. This idea does not conflict with earlier ideas about glutamate-induced $Ca^{2+}$ entry as a causative event in cell death. Both could be required simultaneously. Alternatively, the protective effect of NMDA receptor antagonists may be explained by blockade of $Zn^{2+}$ transport through the receptor ion channel[6]. This is reminiscent of the demonstration that the *in vitro* neurotoxicity of $Pb^{2+}$ can be blocked by NMDA receptor antagonists[16]. The intracellular target for $Zn^{2+}$ is not known. $Zn^{2+}$ reacts with protein thiols and cell death could result from generalized disruption of enzyme pathways without there necessarily being any one single target.

**Parkinson's disease and $Fe^{2+}$**

Iron is another metal with a multitude of normal functions in biology, but which appears under certain circumstances to contribute to neuronal cell death[17]. Free ferrous ions can reduce $H_2O_2$ to produce hydroxyl radicals (Fenton reaction). At a slower rate, ferric chelates can also convert $H_2O_2$ to OH·. This most damaging of reactive oxygen species is normally prevented from forming by the action of catalase converting $H_2O_2$ to oxygen and water. The key to preventing iron toxicity is therefore to ensure that iron is sequestered in such a way as to prevent it taking part in Fenton chemistry. Iron in the body is complexed in haemoproteins, other metalloenzymes, and iron transporting and storage proteins such as transferrin and ferritin. These chelates are poor Fenton reagents, but some can be induced to release small quantities of iron following exposure to reactive oxygen species such as $O_2^{\cdot -}$ or $H_2O_2$[18]. The potential therefore exists for iron released locally in response to oxidative stress to participate in Fenton chemistry leading in turn to further protein damage.

The neurodegenerative disease in which iron has been most strongly implicated is Parkinson's disease[19]. This disease is characterised by selective and progressive loss of dopaminergic neurones projecting from the substantia nigra to the striatum. The oxidative deamination of dopamine by monoamine oxidase leads to the production of $H_2O_2$ which, as described above, could lead to the production of toxic hydroxyl radicals. There have been many reports that the iron content of parkinsonian substantia nigra pars compacta is elevated[20]. This in itself would not be relevant if normal defensive mechanisms were in operation. However, while iron overload normally induces expression of ferritin, in Parkinson's disease brain, ferritin levels are reduced not increased. Furthermore, other protective mechanisms seem to be deficient[21]. Catalase, glutathione and ascorbate are reduced[21] and increased levels of malondialdehyde[22] have been detected. This is a marker of lipid peroxidation, providing evidence that Parkinson's brain tissue is subject to attack by free radical species. The already unfavourable conditions may be further exacerbated by the presence of melanin which arises in these dopaminergic neurones from oxidation of dopamine. Melanin binds iron with high affinity and promotes iron-dependent lipid peroxidation suggesting that the complex is a good Fenton reagent[19]. The involvement of iron in Parkinson's disease is not incompatible with ideas that the disease could be caused by an endogenous neurotoxin. Conditions in these neurones appear to be such that they are vulnerable to radical-mediated degeneration from whatever initial cause and that iron may play an important role in the process.

**Bipolar affective disorder, dorsoventral patterning and $Li^+$**

Lithium salts have been used now for many years for the effective treatment of bipolar affective disorder or manic depression[7]. More recently, other drugs have been found to be effective - valproate and carbamazepine, for example[23] - but this has not shed any light on what is still a poorly understood disease. The mechanism of lithium itself is still controversial. Since therapeutic plasma levels of $Li^+$ are about 0.5-1.4mM[24] and the intracellular concentrations in neurones are unknown but could be greater, one could pick from any number of processes which are affected by $Li^+$ in the mM range. $Li^+$ mimics both $Na^+$ and $Mg^{2+}$ and therefore is potentially capable of modulating many enzymes and transporters. $Li^+$ is rather toxic at plasma levels two to three times the therapeutic level[24], presumably as a result of inhibiting some other key process or processes. The question is therefore whether there is a single target for the beneficial action of $Li^+$ and how to identify it. Over recent years, much attention has focused on the ability of $Li^+$ to affect phosphoinositide signalling[7]. The evidence is all circumstantial, but the hypothesis has attractive features in that it can explain the selectivity of the drug through action on particular populations of overactive neurones[25]. The key discovery was that the enzyme inositol monophosphatase was quite sensitive to inhibition by $Li^+$ (Ki of around 0.1mM), and that the mode of inhibition, unusually, was uncompetitive[26]. The enzyme is important in phosphoinositide signalling since it is responsible for regenerating inositol for phosphoinositide synthesis (Fig.2). That is, receptor-mediated breakdown of polyphosphoinositides to give inositol polyphosphate (IP3 and IP4) second messengers and diacylglycerol would cease unless the inositol can be recycled[25]. Inositol monophosphatase is involved both in the recycling and also in *de novo* synthesis from glucose. and the only other source of inositol is therefore that which can be transported into the cell from outside sources (dietary, for example). The attraction of the uncompetitive mode of inhibition of the enzyme by $Li^+$ lay in the unusual property that uncompetitive inhibitors have less effect on enzyme activity and metabolite levels when the enzyme is working at low capacity i.e. not saturated by its substrate, than when it is working at higher capacity.

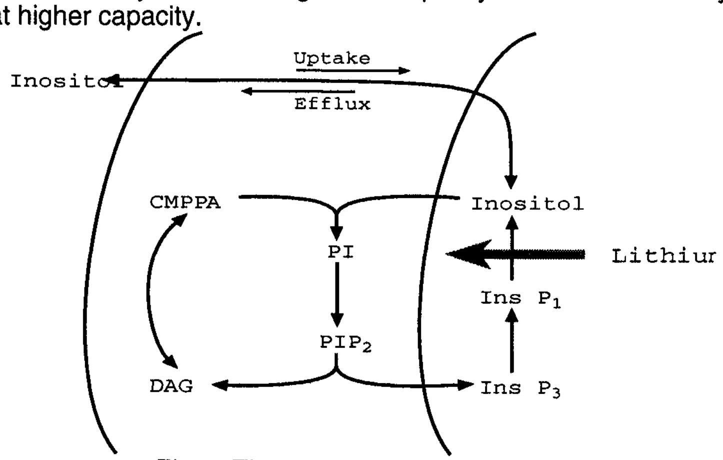

Fig.2. The phosphoinositide cycle

This means that $Li^+$ might be expected to have little effect on metabolite levels of the phosphoinositide cycle when the cycle is not being stimulated by agonist action. However, in the presence of an agonist, substrates (inositol monophosphates) would increase and inositol would decrease. The former has been shown repeatedly in many *in vitro* cell systems[27] and *in vivo* in rat[28] and mouse[29].

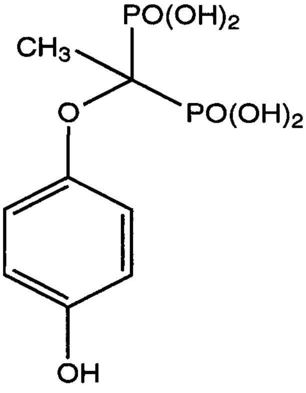

Fig.3 Structure of L-690,330

Our own work on the discovery of selective organic inhibitors of inositol monophosphatase confirmed that the effects of $Li^+$ on phosphoinositide cycle intermediates *in vitro* and *in vivo* could be explained by underlying inhibition of this enzyme. Prodrugs of the inhibitor L-690,330 (Fig.3) had very potent $Li^+$-like effects on inositol phosphate levels (IP1, IP3 and IP4) in a cell line[30], and L-690,330 itself was capable of elevating IP1 in mouse brain[31].

The question that has proven much harder to answer is whether inositol depletion occurs to any extent. The consequences of inositol depletion would be a reduced rate of synthesis of phosphoinositides resulting in reduced capacity for production of IP3 and IP4. This has been shown *in vitro* in brain slices or cell lines in which the depletion of inositol also gives rise to a marked increase in the other substrate for phosphoinositide synthesis, CMPPA[32]. By a reversal of the normal reaction, this can lead to an increase in diacylglycerol. *In vivo*, the evidence that $Li^+$, at therapeutic levels, causes decreased cell signalling through this pathway is much harder to find. Marked elevation of inositol monophosphates and a reciprocal decrease in brain inositol requires high doses of $Li^+$ and simultaneous treatment with an agonist, such as pilocarpine[28]. It can be argued that since the effect is really only seen at high doses then this is more likely to be related to the toxicity of $Li^+$ than its therapeutic effect. The extent of the effect on the phosphoinositide cycle depends not only on the extent to which $Li^+$ is elevating inositol monophosphates but also on the extent to which the cell can restore its inositol level through transport. This is controlled by a balance between uptake and efflux pathways[25]. Since this balance can vary from cell to cell, some cells are more vulnerable to inositol depletion than others and it may therefore be only a subset of neurones which are affected by $Li^+$. The remainder, despite inhibition of inositol monophosphatase can limit the effect by maintaining intracellular inositol through transport while others cannot, and signalling is decreased. The appeal of this theory is that it offers an explanation for the *in vivo* selectivity of $Li^+$, which by the standards of organic drugs, is a very unselective ligand. To find further evidence for inositol depletion, various outcomes of $Li^+$ *in vivo* have been examined for their prevention by exogenous inositol. For example, the potentiating effect of $Li^+$ on pilocarpine-induced seizures is prevented by prior adminstration of *myo*-inositol i.c.v[33]. The belief that selective action by *myo*-inositol (the isomer which is incorporated into phophoinositides) as opposed to *scyllo*-inositol (which is not) provides adequate proof of the mechanism, was damaged by the demonstration that *epi*-inositol could also prevent seizures[34]. *Epi*-inositol is not a substrate for phosphoinositide synthesis. Very recently, another effect of $Li^+$ which was prevented by *myo*-inositol and thought therefore to be linked to the phosphoinositide cycle, was shown to be caused by quite a different mechanism.

Lithium salts cause dorsalisation of Xenopus embryos, that is, duplication of anterior structures such as the head, eyes and neural tube, at the expense of posterior structures such as the tail[35]. A number of genes are implicated in dorsoventral patterning in vertebrates including a family of glycoproteins[36] which include Wnt-1 in mouse and Xwnt-8 in Xenopus. Work in Drosophila has established that one of the genes involved in the wingless (equivalent to Wnt-1) signalling pathway (shaggy or zw-3) is the homologue of a mammalian protein kinase, glycogen synthase kinase 3[37]. Injection of Xenopus embryos with dominant negative mutants of GSK-3 mimics the phenotype of $Li^+$ treatment i.e. loss of GSK-3 leads to dorsalisation, while overexpression of the enzyme leads to ventralisation[38]. The evidence that the effect of $Li^+$ was via inhibition of inositol monophosphatase was shown to be incorrect, since the specific inhibitor, L-690,330, did not cause dorsalisation despite complete inhibition of the enzyme in the embryos[39]. In fact, $Li^+$ is an uncompetitive inhibitor of GSK-3, with a Ki in the mM range[39]. While not as potent an inhibitor of GSK-3 as inositol monophosphatase, inhibition would occur at therapeutic plasma concentrations of $Li^+$[40]. So, is GSK-3 a plausible candidate for the action of $Li^+$ in the treatment of psychiatric disorder? The enzyme has a number of functions in addition to regulating glycogen synthesis. It is certainly involved in insulin signalling and is itself regulated by a number of kinases via ser/thr phosphorylation. Parenthetically, it is also known as tau protein kinase 1 and has been suggested to play a role in Alzheimer's disease through mediating the cytotoxicity of the amyloid-( peptide[41]. However, there is no answer to this question other than to test a selective inhibitor in the clinic.

**Oxidative stress, neurodegeneration and organomanganese complexes**

Reference has already been made to the possible role of reactive oxygen species in neurodegeneration[17]. In stroke or other conditions leading to excessive glutamate release, the influx of $Ca^{2+}$ ions through the NMDA receptor can lead to activation of systems producing such species (mitochondrial dysfunction, xanthine oxidase activation) and free radical scavenging may be a valid approach to therapy. There was great interest therefore in the aminosteroid "lazaroid", tirilazad, from Upjohn, which showed excellent levels of neuroprotection in animal models of traumatic brain damage[42]. Tirilazad is approved for the treatment of subarachnoid haemorrhage[43], but results in head injury and in stroke have not s open the question whether it is the concept or the drug that is lacking.

Another approach which may help provide an answer is that of the organomanganese complexes being developed by Eukarion. These compounds are able to catalyse dismutation of superoxide to $H_2O_2$ and $O_2$ as well as catalysing breakdown of $H_2O_2$ to $O_2$ and water[8]. They therefore act as small molecule superoxide dismutases and catalases. The relative activity of the two is determined by the exact nature of the organic chelator. One of these compounds, EUK-8 (Fig.4), has been tested in a number of model systems in which free radical species have been implicated as the toxic agents.

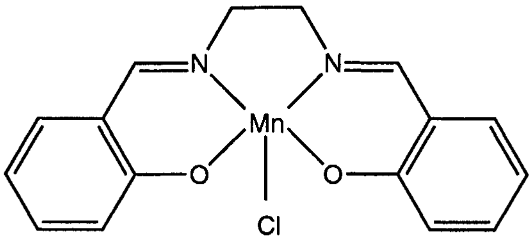

Fig. 4. Structure of EUK-8

In the neuroscience area, EUK-8 has been shown to prevent the deterioration of synaptic activity in rat hippocampal slices subjected to hypoxia and acidosis, a possible indicator of activity in the treatment of stroke[45]. *In vivo* central activity of EUK-8 has also been shown in mice following treatment with the neurotoxins, MPTP and 6-hydroxydopamine[46]. These agents cause selective loss of dopaminergic neurones and MPTP in particular is used to generate a primate model of Parkinson's disease. The similarity between drug-induced and idiopathic parkinsonism has done much to fuel speculation that an environmental neurotoxin may be a key factor in the disease. However, these two toxins are known to cause cell death through a free-radical mediated process and systemic or intracerebrovascular EUK-8 provided protection against MPTP and 6-hydroxydopamine respectively[46].

More controversial by far is the idea that the amyloid-( peptide is the direct cause of neuronal death in Alzheimer's disease and that it does so through a free-radical process. Familial Alzheimer's disease results from mutations in either the amyloid precursor protein, APP, or in either of the two presenilins 1 and 2 (PS1 and PS2)[47]. Despite any good understanding of the normal role of APP or the presenilins, or how mutation of the latter proteins affects the processing of the former, it is now clear that all the mutations lead to altered APP processing such that there is either an increased production of A( peptides in general or a selective increase in the longer 42-43 aminoacid form. The latter is more prone to aggregate into a state which is toxic to cells *in vitro* and this therefore provides an explanation for early onset Alzheimer's disease. The cause of sporadic Alzheimer's disease of course, is not explained by this mechanism even though A( may be the toxic agent. Moreover, despite a huge effort from many laboratories, there is no concensus on how A( kills cells. Certainly, reactive oxygen species have been proposed as the culprits and antioxidants have some efficacy in preventing acute cell death caused by A( *in vitro*[48]. EUK-8 is one of these and provides protection against A(-induced cell death in organotypic hippocampal cultures, preventing cell lysis and reducing levels of reactive oxygen species[49]. It is difficult to know how good a model of Alzheimer's disease this is, and in the absence of an animal model with Alzheimer-like neuronal degeneration, the efficacy of this and similar drugs will have to be tested in the clinic.

### Conclusions

The use of the simple cation, $Li^+$, as a therapeutic agent is unique and it is hard to see what lessons can be learned from it, particularly as its site of action is still not understood. Since the most plausible mechanisms at present involve action on intracellular signalling pathways, $Li^+$ may be a pointer to the potential of drugs acting on these pathways as an alternative to the current reliance on cell surface receptor agonists and antagonists. This being said, the justification for metal binding sites as a good means of modulating protein function is not clear, certainly while we do not understand exactly how $Li^+$ acts. By modern standards, $Li^+$ would not be an acceptable drug, having a very poor therapeutic index. Careful use has allowed it to become a successful drug, but the toxicity is a real problem which may be mechanism-based but may also be a reflection of lack of specificity of $Li^+$ for the metal binding site of its target protein. In the same way, the toxicity of divalent and trivalent metal ions is likely to be exerted on a wide range of different targets and intervention strategies should reflect this. Metal-chelation approaches therefore have merit in that they avoid the need to identify the molecular targets of the toxic metal ion, and would also be effective in circumstances where the toxicity is indirect, for example, where production of free radical species is the basis of the disorder. Metal chelators have been shown to have effects in animal models in preventing neurodegeneration. In an example given above, CaEDTA prevented cell death in a stroke model. The iron chelator, desferrioxamine i.c.v, prevents loss of dopaminergic neurones following administration of 6-hydroxydopamine to rat[50]. The same compound is effective orally in reducing aluminium in brain and peripheral tissues of aluminium-loaded rats[51] and desferrioxamine is used to treat aluminium-related osteomalacia and dialysis dementia in renal dialysis patients. However, evidence for efficacy of chelation strategies in major neurodegenerative disease is much less convincing. The epidemiological evidence for aluminium as a risk factor for Alzheimer's disease as well as the evidence for aluminium as a causative factor in dialysis dementia prompted a clinical trial of desferrioxamine in this disease, the results of which were unconvincing[52].

An alternative way to chelate metals more effectively would be to increase expression of natural chelators. Reference has already been made above to the reduction in ferritin in Parkinson's disease brain. Interest has also centred around the metallothioneins, in particular, MT-III, which is expressed in the glutamatergic pyramidal cells of the hippocampus and granule cells of the dentate gyrus among others[53]. It has been proposed that MT-III is involved in zinc homeostasis and indirectly therefore in modulating glutamatergic transmission. A role for MT-III in Alzheimer's diease and other neurodegenerative disorders remains controversial, but the idea that manipulating expression of MT's might be an approach to neuroprotection is intriguing.

### References

1. Winegar, B.D. and Lansman, J.B. *J. Physiol. (London),* 1990, **425**:563.
2. Winegar, B.D., Kelly, R. and Lansman, J.B. *J. Gen. Physiol.,* 1991, **97**:351.
3. Weiss, J.H., Hartley, D.M., Koh, J.Y. and Choi, D.W. *Neuron,* 1993, **1**:43.
4. Westbrook, G.L. and Mayer, M.L. *Nature,* 1987, **328**:640.
5. Koh, J.Y. and Choi, D.W. *Neurosci.*, 1994, **60**:1049.
6. Koh, J.Y., Sang, W.S., Byoung, J.G., Yong, Y.H., Chung, Y.H. and Choi, D.W. *Science*, 1996, **272**:1013.
7. The Lithium Mechanisms Study Group. *Rev. Contemp. Pharmacother.*
8. Baudry, M., Etienne, S., Bruce, A., Palucki, M., Jacobsen, E. and Malfroy, B. *Biochem. Biophys, Res. Commun.*, 1993, **192**:964.
9. Hollman, M and Heinneman, S. *Ann. Rev. Neurosci.*, 1994, **17**:31.
10. Nowak, L., Bregestovski, P., Herbert, A. and Prochiantz, Z. *Nature*, 1984, **307**:462.
11. Koroshetz, W.J. and Moskowitz, M.A. *Trends. Pharm. Sci.*, 1996, **17**:227.
12. Mayer, M.L. and Miller, R.J. *Trends Pharm. Sci.*, 1990, **11**:254.
13. Dugan, L.L., Sensi, S.L., Canzoniero, L.M., Handran, S.D., Rothman, S.M., Lin, T.S., Goldberg, M.P. and Choi, D.W. *J. Neurosci.*, 1995, **15**:6377.
14. Patt, A., Harken, A.H., Burton, L.K., Rodell, T.C., Piermattei, D., Schorr, W.J., Parker, N.B., Berger, E.M., Horesh, I.R., Terada, L.S., Linas, S.L., Cheronis, J.C. and Repine, J.E. *J. Clin. Invest.*, 1988, **81**: 1556.
15. Tonder, N., Johansen, F.F., Frederickson, C.J., Zimmer, J. and Diemar, N.H. *Neurosci. Lett.*, 1990, **109**:247.
16. Perovic, S., Pergande, G., Ushijima, H., Kelve, M., Forrest, J., Muller, W.E. *Neurodegen.*, 1995, **4**:369.
17. Gutteridge, J.M.C. *Ann. N.Y. Acad. Sci.*, 1994, **738**: 201.
18. Biemond, P., Van Eijk, H.G., Swaak, A.J.G. and Koster, J.F. *J. Clin. Invest.*, 1984, **73**:1576.
19. Ben-Shachar, D. and Youdim, M.B.H. *Prog. Neuro. Psychopharmacol. Biol. Psych.*, 1993, **17**:139.
20. Dexter, D.T., Carayon. A., Javoy-Agid, F., Agid, Y., Wells, F.R., Daniel, S.E., Lees, A.J., Jenner, P and Marsden, C.D. *Brain*, 1991, **114**:1953.

21. Riederer, P., Sofic, E., Rausch, W.D., Schmidt, B., Reynolds, G.P., Jellinger, K., and Youdim, M.B.H. *J. Neurochem.*, 1989, **52**:515.
22. Dexter, D.T., Carter, C.J., Wells, F.R., Javoy-Agid, F., Agid, Y., Lees, A., Jenner,P. and Marsden, C.D. *J. Neurochem.*, 1989, **52**:381.
23. Bowden, C.L., Brady, K.T., Forster, P.L., Goodwin, F.K. and Tohen, M. *J. Clin. Psych.*, 1996, **57**:1.
24. *The Merck Manual of Diagnosis and Therapy* (Berkow, R. and Fletcher, A.J., eds), Merck Sharp and Dohme Research Laboratories, 15th Edn., p.2413.
25. Nahorski, S.R., Ragan, C.I. and Challiss, R.A.J. *Trends Pharm. Sci.*, 1991, **12**:297.
26. Hallcher, L.M. and Sherman, W.R. *J. Biol. Chem.*, 1980, **255**:10896.
27. Kennedy, E.D., Challiss, R.A.J., Ragan, C.I. and Nahorski, S.R. *Biochem. J.*, 1990, **267**:781.
28. Sherman, W.R., Gish, B.G., Honchar, M.P. and Munsell, L.Y. *Fed. Proc.*, 1986, **45**:2639.
29. Atack, J.R., Cook, S.M., Watt, A.P. and Ragan, C.I. *J. Neurochem.*, 1992, **59**:1946.
30. Atack, J.R., Prior, A.M., Griffith, D. and Ragan, C.I. *Br. J. Pharmacol.*, 1993, **110**:809.
31. Atack, J.R., Cook, S.M., Watt, A.P., Fletcher, S.R. and Ragan, C.I. *J. Neurochem.*, 1993, **60**:652.
32. Downes, C.P. and Stone, M.A. *Biochem. J.*, 1986, **234**:199.
33. Tricklebank, M.D., Singh, L., Jackson, A. and Oles, R.J. *Brain Res.*, 1991, **558**:145.
34. Williams, J.R. and Jope, R.S. *Brain. Res.*, 1995, **685**:169.
35. Busa, W.B. and Gimlich, R.L. *Devel. Biol.*, 1989, **132**:315.
36. Nusse, R. and Varmus, H.A. *Cell*, 1992, **69**:1073.
37. Siegfried, E., Chou, T.B. and Perrimon, N. *Cell*, 1992, **71**:1167.
38. Xi, H., Saint-Jeannet, J.P., Woodgett, J.R., Varmus, H.E. and Dawid, I.B. *Nature*, 1995, **374**:617.
39. Klein, P.S. and Melton, D.A. *Proc. Natl. Acad. Sci. U.S.A.*, 1996, **93**:8455.
40. Stambolic, V., Ruel, L. and Woodgett, J.R. *Current Biol.*, 1996, **12**:1664.
41. Takashima, A., Noguchi, K., Michel, G., Mercken, M., Hoshi, M., Ishiguro, K. and Imahori, K. *Neurosci. Lett.*, 1996, **203**:33.
42. Clark, W.M., Hazel, J.S. and Coull, B.M. *Drugs*, 1995, **50**:971.
43. Hall, E.D., *Eur. J. Anaesthesiol.*, 1996, **13**:279.
44. The RANTTAS Investigators. *Stroke*, 1996, **27**:1453.
45. Musleh, W., Bruce, A., Malfroy, B. and Baudry, M. *Neuropharmacol.*, 1994, **33**:929.
46. Bruce, A., Musleh, W., Malfroy, B. and Baudry, M. *Abstr. Soc. Neurosci.*, 1993, 686.18.
47. Scheuner, D., Eckman, C., Jensen, M., Song, X., Citron, M., Suzuki, N., Bird, T.D., Hardy, J., Hutton, M., Kukull, W., Larson, E., Levy-Lahad, E., Viitanen, M., Peskind, E., Poorkaj, P., Schellenberg, G., Tanzi, R., Wasco, W., Lannfelt, L., Selkoe, D. and Younkin,S. *Nature Med.*, 1996, **2**:864.
48. Friedlich, A.L. and Butcher, L.L. *Neurobiol. Aging*, 1994, **15**:443.
49. Bruce, A.J., Malfroy, B. and Baudry, M. *Proc. Natl. Acad. Sci. U.S.A.*, 1996, **93**:2312.
50. Ben-Shachar, D., Eshel, G., Riederer, P. and Youdim, M.B.H. *Ann. Neurol.*, 1992, **32**:5105.
51. Florence, A.L., Gauthier, A., Ward, R.J. and Crichton, R.R. *Neurodegen.*, 1995, **4**:449.
52. Crapper McLachlan, D.R., Dalton, A.J., Kruck, T.P., Bell, M.Y., Smith, W.L., Kalow, W. and Andrews, D.F. *Lancet*, 1991, **337**:1304.
53. Aschner, M. *FASEB J.*, 1996, **10**:1129.

